# Effects of Rice Root Development and Rhizosphere Soil on Methane Emission in Paddy Fields

**DOI:** 10.3390/plants13223223

**Published:** 2024-11-16

**Authors:** Sheng Guan, Zhijuan Qi, Sirui Li, Sicheng Du, Dan Xu

**Affiliations:** 1 School of Water Conservancy and Civil Engineering, Northeast Agricultural University, Harbin 150030, China; 18904602113@163.com (S.G.); zhijuan.qi@neau.edu.cn (Z.Q.); 13945358017@163.com (S.L.); dusicheng@neau.edu.cn (S.D.); 2 Key Laboratory of Effective Utilization of Agricultural Water Resources, Ministry of Agriculture and Rural Affairs, Northeast Agricultural University, Harbin 150030, China; 3 College of Arts and Sciences, Northeast Agricultural University, Harbin 150030, China

**Keywords:** methane, rice root development, rhizosphere soil, methanogens, methanotroph

## Abstract

Paddy fields are important anthropogenic emission sources of methane (CH_4_). However, it is not clear how rice root development and rhizosphere soil properties affect CH_4_ emissions. Therefore, we selected rice varieties with similar growth periods but different root traits in the local area. We measured CH_4_ emission fluxes, cumulative CH_4_ emissions, root dry weight, root length, and the dissolved organic carbon (DOC), microbial biomass carbon (MBC), redox potential (Eh), ammonium nitrogen (${NH}_{4}^{+}$–N), and nitrate nitrogen (${NO}_{3}^{-}$–N) contents in rhizosphere soil. Methanogens and methanotrophs are crucial factors influencing CH_4_ emissions; thus, their abundance and community composition were also assessed. The result showed that CH_4_ fluxes of each rice variety reached the peak at tillering stage and jointing-booting stage. The CH_4_ emissions in tillering stage were the largest in each growth period. CH_4_ emissions had negative correlations with root length, root dry weight, Eh ${NO}_{3}^{-}$–N, methanotroph abundance, and the *pmoA*/*mcrA* ratio, and positive correlations with ${NH}_{4}^{+}$–N, MBC, DOC, and methanogen abundance. Path analysis confirmed methanogens and methanotrophs as direct influences on CH_4_ emissions. Root development and rhizosphere soil properties affect CH_4_ emissions indirectly through these microbes. This study suggests that choosing rice varieties with good root systems and managing the rhizosphere soil can effectively reduce CH_4_ emissions.

## 1. Introduction

In light of the critical scenario of global warming, achieving the objective of constraining temperature increases to within 1.5 °C demands a substantial reduction in greenhouse gas emissions [1]. Methane (CH_4_), the second most prevalent greenhouse gas after carbon dioxide (CO_2_), constitutes 16% of anthropogenic greenhouse gas emissions [2]. It is worth noting that the contribution of CH_4_ to the greenhouse effect is about 20%, and the warming potential of CH_4_ is 28 on the 100-year scale [3]. Paddy fields constitute an important anthropogenic source of CH_4_. Consequently, reducing CH_4_ emissions from paddy fields is of great significance for alleviating the global greenhouse effect.

The disparity in CH_4_ emissions among various rice cultivars can be as significant as a six-fold difference [4]. Research had indicated that this difference is attributable to the distinct activities of methanogens and methanotrophs present in the rhizosphere of different rice varieties [5]. In the flooded anaerobic environment, methanogens utilize the organic matter secreted either by rice roots or the soil organic carbon as metabolic substrates, and the heightened methanogen activity fosters the generation of CH_4_ [6]. Methanotrophs harness CH_4_ as a carbon and energy source, oxidizing 60~70% of the CH_4_ present in the rhizosphere soil prior to its release into the atmosphere [7]. This process plays a pivotal role in curtailing the net emissions of CH_4_. Despite the acknowledged influence of methanogens and methanotrophs on CH_4_ emissions from paddy fields, the precise regulatory mechanisms through which rice roots and rhizosphere soils affect these microbial activities remain unclear. Therefore, an in-depth study of how rice roots and rhizosphere soil affect CH_4_ emissions through methanogens and methanotrophs is crucial for CH_4_ emission reduction in paddy fields.

Studies have shown that rice roots have a deeper effect on CH_4_ emissions than aboveground parts [8,9,10]. As an important organ of rice, rice roots not only fix rice plants and are responsible for the absorption of water and nutrients, but also provide a link between the rice and CH_4_ emission-related microorganisms in the soil. Studies have shown that planting rice varieties with better root development can reduce CH_4_ emissions from paddy fields [11]. The reason is that higher root biomass can provide a suitable environment for the oxidation process of CH_4_ by methanotrophs [12]. In addition, about 90% of the CH_4_ oxidation process occurs in the deeper soil layer [13]. There has been observed a significant negative correlation between CH_4_ emissions and rice roots, so the selection of rice varieties with large root distribution can reduce CH_4_ emissions from paddy fields [14]. A study had demonstrated that rice cultivars boasting a greater root biomass tend to exhibit enhanced rates of root exudation [15]. Root exudates serve as substrates for methanogenic bacteria [16] and, as such, CH_4_ emissions from paddy fields are closely related to rice root development. Thus, it is of great scientific value and practical significance to explore the mechanism by which rice root development impacts methanogens and methanotrophs for the effective control of CH_4_ emissions in paddy fields.

The rhizosphere soil is the soil part directly affected by plant roots, in which microorganisms show higher activity. The composition of rhizosphere soil has an important impact on methanogens and methanotrophs. On the one hand, soil active organic carbon, including dissolved organic carbon (DOC) and microbial biomass carbon (MBC), is an important factor affecting CH_4_ production capacity [17,18]. Their content directly affects the activity and community structure of methanogens, which in turn affects the production of CH_4_ in the paddy fields [19,20]. On the other hand, soil organic carbon can also promote the activity and abundance of methanotrophs, thus increasing the oxidation potential of CH_4_ in paddy fields [21]. Moreover, ammonium nitrogen (${NH}_{4}^{+}$–N) and nitrate nitrogen (${NO}_{3}^{-}$–N) in the rhizosphere soil have a regulatory effect on the activity of methanogens and methanotrophs. ${NH}_{4}^{+}$–N had been found to enhance the activity of methanotrophs; meanwhile, ${NO}_{3}^{-}$–N inhibits the activity of methanogens and promotes the activity of methanotrophs, resulting in reduced CH_4_ emissions [22,23]. However, other studies suggested that ${NH}_{4}^{+}$–N and ${NO}_{3}^{-}$–N correlate positively with methanogen and negatively with methanotroph abundance, potentially promoting the CH_4_ emissions from the rice fields [24]. And the study showed that Eh directly determines the amount and rate of CH_4_ production in soil, and also causes morphological and physiological changes in rice plants, affecting the gas exchange between soil and atmosphere [25]. It is also important to study the effect of Eh on CH_4_ emissions. In summary, the effects of rhizosphere soil properties on methanogens and methanotrophs need to be further studied.

In Northeast China, the black soil area is one of the four major areas of black soil in the world [26,27,28]. This area has a high soil organic matter content and is the most fertile area in China [29]. The rice planting area in Northeast China reaches 5.62 × 10^6^ ha [30], and provides an important rice supply base. However, it is evident that the ongoing increase in greenhouse gas emissions, particularly CH_4_, could present a risk to rice production in this region [31]. Therefore, in order to alleviate the global greenhouse effect, it is necessary to conduct in-depth research on the CH_4_ emission mechanism in Northeast China.

Previous studies had focused on the differences between methanogens and methanotrophs in different rice varieties [5,32]. The aim of this study lies in selecting rice varieties with different root characteristics and exploring the effects of rice roots and rhizosphere soil properties on CH_4_ emissions in paddy fields by analyzing the relationship between rice root development, rhizosphere soil properties, and target microbial communities (methanogens and methanotrophs). In this study, we measured and analyzed CH_4_ emissions, root development, rhizosphere soil indexes (DOC, MBC, ${NH}_{4}^{+}$–N, ${NO}_{3}^{-}$–N, Eh), and the abundance of methanogens and methanotrophs in five selected local representative rice varieties through field experiments. In addition, we explored the mechanism of rice root characteristics on CH_4_ emissions by path analysis. This analytic method shows how rice roots and rhizosphere soil affect methanogens and methanotrophs directly and indirectly and thus affect CH_4_ emissions from paddy fields.

## 2. Results

### 2.1. Difference Analysis of CH_4_ Emissions and Yield of Different Rice Varieties

#### 2.1.1. CH_4_ Emission Fluxes

**Figure 1:**
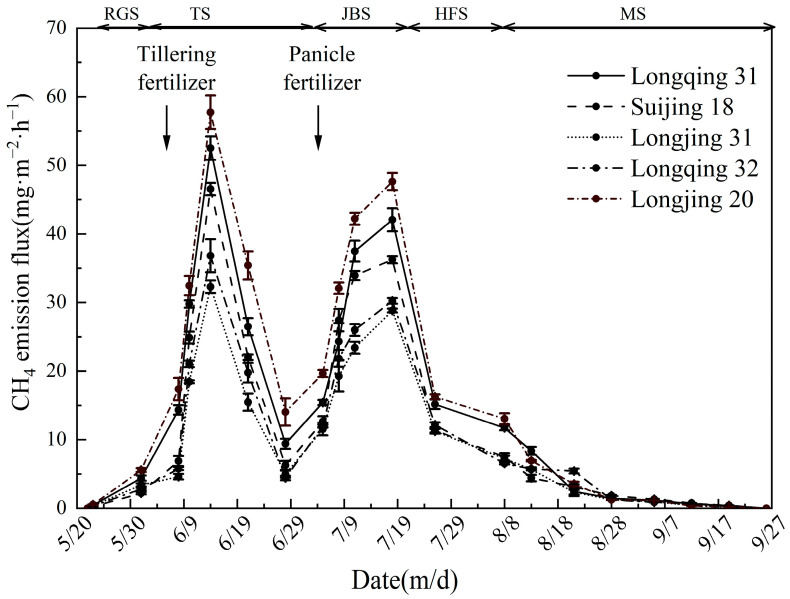
Time course changes in CH_4_ emission fluxes in different rice varieties’ cultivation. Error bar indicates standard deviation (*n* = 3). Growth stages according to BBCH (Biologische Bundesanstalt, Bundessortenamt and Chemical industry) scale [33]: RGS, returning green stage (BBCH 14–19); TS, tillering stage (BBCH 20–29); JBS, jointing–booting stage (BBCH 30–49); HFS, heading–flowering stage (BBCH 51–69); MS, maturity stage (BBCH 71–89). Same as below.

Figure 1 shows the CH_4_ emission fluxes from five distinct rice varieties throughout the growth period. All five rice varieties can be observed exhibiting a double peak. At the initial stage of rice growth, the CH_4_ emission fluxes were observed to be at a relatively low level. As the rice progresses to the returning green stage (the rice plant starts to regain its vitality and turn green once again after transplantation), the CH_4_ emission fluxes exhibited a gradual increase. At the tillering stage, the emission fluxes reached the first peak, with Longjing 31 exhibiting the lowest CH_4_ emission fluxes of 32.3 mg·m^−2^·h^−1^ during this stage. This is 12.3~44.1% lower than that observed in the other varieties. The CH_4_ emission fluxes declined significantly in the late tillering stage with the implementation of sunning management practices. At the end of the sunning period, the CH_4_ emissions climbed rapidly again with the resumption of irrigation activities and the application of fertilizers, and reached a second emission peak. At this stage, the CH_4_ emission fluxes of Longjing 31 remained the lowest, at 28.8 mg·m^−2^·h^−1^, which is 4.7~39.5% lower than that of the other varieties. At the subsequent phases of rice growth, the CH_4_ emission fluxes exhibited a gradual decline, and ultimately stabilize at a lower emission level at the maturity stage.

#### 2.1.2. Cumulative CH_4_ Emissions, Rice Yield, and CH_4_ Emissions per Yield of Each Rice Variety

**Figure 2:**
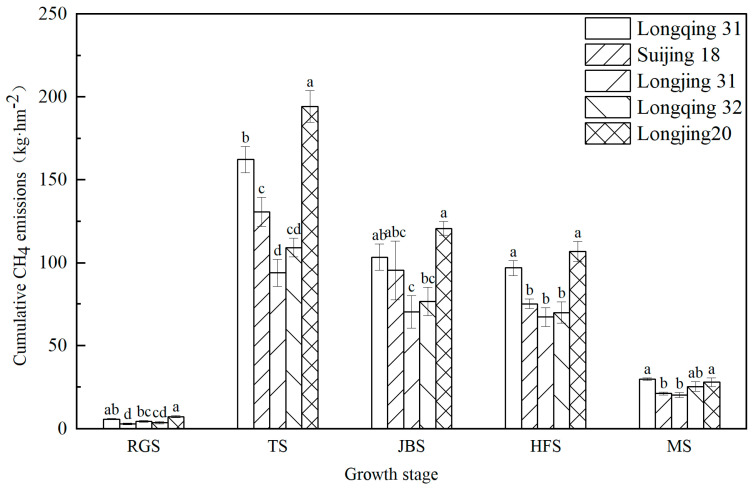
The cumulative CH_4_ emissions at each growth stage in different rice varieties’ cultivation. The different letters in the same growth stage indicate significant differences among the rice varieties (*n* = 3, *p* < 0.05). The error bar indicates the standard deviation.

The cumulative CH_4_ emissions at each growth stage (Figure 2) show that Longjing 20 had the highest emission (7.3 kg·hm^−2^–194.3 kg·hm^−2^) while Longjing 31 had the lowest emission flux (4.52 kg·hm^−2^–93.9 kg·hm^−2^). The tillering stage accounted for the largest emission ratio of 36.6~42.5%, followed by the jointing–booting stage and the heading–flowering stage, accounting for 26.0~29.3% and 23.1~26.2%, respectively. The CH_4_ emissions of 0.9~1.8% in the returning green stage accounted for the lowest proportion in the whole growth period.

The total cumulative CH_4_ emissions, rice yield, and CH_4_ emissions per yield of different rice varieties are shown in Table 1. Significant differences were in total cumulative CH_4_ emissions among the rice varieties (*p* < 0.05). The total cumulative CH_4_ emissions were ranked from low to high as follows: Longjing 31 < Longqing 32 < Suijing 18 < Longqing 31 < Longjing 20. The lowest cumulative CH_4_ emissions were observed in Longjing 31 (256.3 kg·hm^−2^), lower than the other varieties ranging from 10.1 to 44.0%. There were significant differences in rice yield and CH_4_ emissions per yield (*p* < 0.05). Longjing 31 had the highest yield and the lowest CH_4_ emissions per yield, indicating that it was a high-yield and low-emission rice variety. Longjing 20 had the highest CH_4_ emissions per yield due to its lowest production and high CH_4_ emission values.

### 2.2. Analysis of Soil Eh

**Figure 3:**
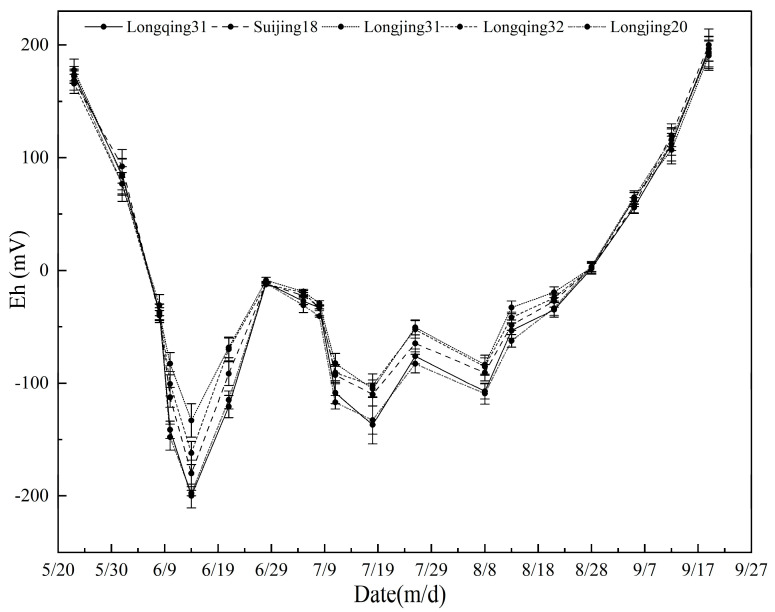
Time course changes in soil Eh value in different rice varieties’ cultivation. Error bar indicates standard deviation (*n* = 3).

The Eh of each rice variety gradually decreased with time from the returning green stage, reached the lowest value in the middle tillering stage, and then increased until the late tillering stage (Figure 3). After entering the jointing–booting stage, the Eh value began to fluctuate until the end of heading–flowering stage. After that, the Eh value rose until the rice matures. The Eh value of Longjing 31, which had a lower CH_4_ emission during the whole growth period, remained at a high level.

### 2.3. Analysis of Root Development

**Figure 4:**
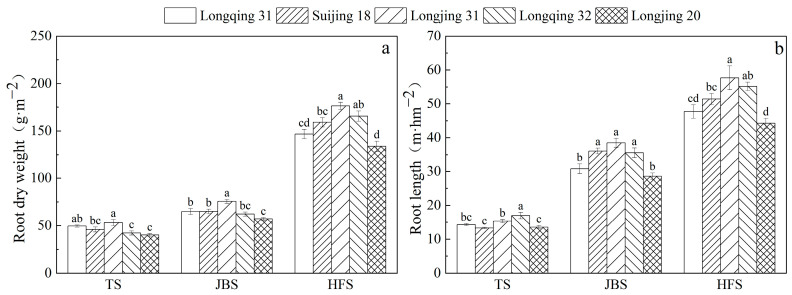
The root dry weight (**a**) and root length (**b**) of different rice varieties at the main growth stages. Different letters in the same growth stage indicate significant differences among the rice varieties (*n* = 3, *p* < 0.05). The error bar indicates the standard deviation.

The development of the rice roots is shown in Figure 4. The growth trends in the root dry weight and root length exhibited a similar pattern, gradually increasing in the rice growth period and reaching a maximum during the heading–flowering stage (Figure 4a). At the tillering and jointing–booting stages, the root dry weights of the five rice varieties were as follows: Longjing 20 < Longqing 32 < Suijing 18 < Longqing 31 < Longjing 31. Longjing 31 had the highest root dry weights of 53.2 g·m^−2^ and 75.7 g·m^−2^ in these two periods. At the heading–flowering stage, the order of root dry weight among rice varieties changed, with the following ranking: Longjing 20 < Longqing 31 < Suijing 18 < Longqing 32 < Longjing 31. At this point, the root dry weight of Longjing 31 was 176.5 g·m^−2^, which was 6.5~31.7% higher than that of the other four varieties (Figure 4a).

At the tillering stage, Longqing 32 demonstrated a root length significantly longer than the other varieties (*p* < 0.05), reaching the length of 17.0 m·hm^−2^, and reflecting an increase of 10.6~25.3% compared to the other varieties (Figure 4b). The root length of Longqing 31 and Longjing 20 was significantly lower than that of other varieties at the jointing–booting stage (*p* < 0.05) (Figure 4b). The root length of Longjing 20 was relatively low among the five varieties in these three periods (Figure 4b).

### 2.4. Analysis of Rhizosphere Soil

**Figure 5:**
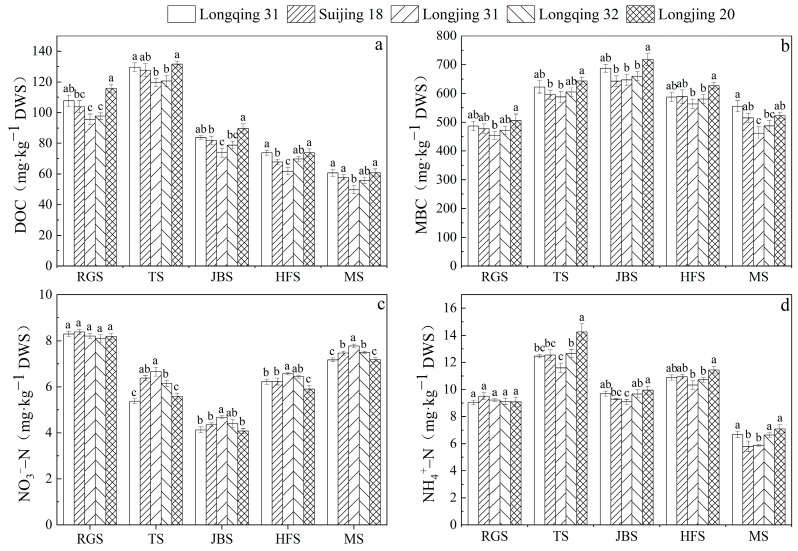
The amount of DOC (**a**), MBC (**b**), ${NO}_{3}^{-}$–N (**c**), and ${NH}_{4}^{+}$–N (**d**) in the rhizosphere soil of different rice varieties at each growth stage. DWS: dry weight of soil. Different letters in the same growth stage indicate significant differences among the rice varieties (*n* = 3, *p* < 0.05). The error bar indicates the standard deviation.

As illustrated in Figure 5, the contents of the different soil components show obvious dynamic change patterns during the rice growth period, which show a commonality under the different rice varieties.

The DOC content showed an increasing trend between the returning green stage and tillering stages, and then gradually decreased until the end of the maturity stage (Figure 5a). The DOC content of Longjing 31 was relatively low at different stages, with specific values of 95.7 mg·kg^−1^ DWS, 119.7 mg·kg^−1^ DWS, 73.8 mg·kg^−1^ DWS, 61.9 mg·kg^−1^ DWS, and 49.6 mg·kg^−1^ DWS, which were lower than the other four varieties at different stages by 2.1~17.2%, 0.8~9.1%, 6.3~17.7%, 8.8~16.2%, and 10.8~16.2% (Figure 5a).

The MBC content demonstrated a gradual increase from the returning green stage until it reached its peak at the jointing–booting stage (Figure 5b). At the returning green stage, tillering, and heading–flowering stages, the MBC content of Longjing 31 was 8.7~10.4% lower than that of Longjing 20. Longjing 20, with the highest MBC content, was 11.6% higher than Suijing 18, with the lowest MBC content, at the jointing–booting stages. At the heading–flowering stage, Longjing 20, with the highest MBC content, was 11.3% higher than Longjing 31, with the lowest content. At the maturity stage, the MBC content changed so that Longqing 31 had the highest content, followed by Longjing 20, then Suijing 18, Longqing 32, and, finally, Longjing 31 had the lowest MBC content (Figure 5b).

The content of ${NO}_{3}^{-}$–N began to decrease at the beginning of the returning green stage and reached the lowest point at the jointing–booting stage (Figure 5c). Thereafter, the ${NO}_{3}^{-}$–N content demonstrated a gradual increase until the plants reached the maturity stage. During the returning green stage, there were no significant differences in the content of ${NO}_{3}^{-}$–N between the different rice varieties (*p* > 0.05). At the heading–flowering stage, Longjing 20 had the lowest ${NO}_{3}^{-}$–N content in this period, which was 5.3% to 10.3% lower than the other varieties. At maturity stage, Longjing 31 was significantly higher than other varieties (Figure 5c).

The ${NH}_{4}^{+}$–N content displayed an upward trajectory followed by a downward one, reaching two peaks at both the tillering and heading–flowering stage (Figure 5d). There was no significant difference in ${NH}_{4}^{+}$–N content among rice varieties at the returning green stage (*p* > 0.05). The ${NH}_{4}^{+}$–N content of Longjing 20 was the highest at the tillering and heading–flowering stages, reaching 14.3 mg·kg^−1^ DWS and 11.5 mg·kg^−1^ DWS, respectively. These values were 12.7–22.9% and 4.7–10.7% higher than the other varieties. At the maturity stage, the differences in the ${NH}_{4}^{+}$–N contents of Longqing 31, Longqing 32, and Longjing 20 were not significant (*p* > 0.05). However, these three varieties of rice exhibited significantly higher ${NH}_{4}^{+}$–N contents than Suijing 18 and Longjing 31 (*p* < 0.05) (Figure 5d).

### 2.5. Abundance and Community Structure of Methanogens and Methanotrophs in Different Rice Varieties

**Figure 6:**
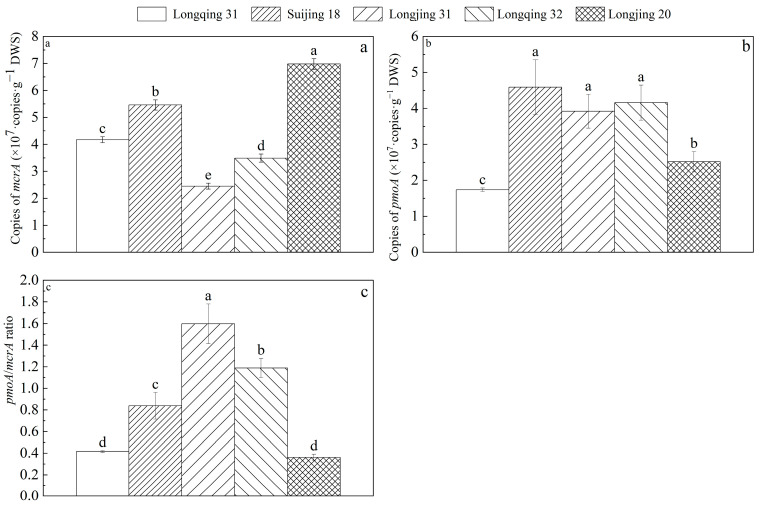
The abundance of the *mcrA* (**a**) and *pmoA* (**b**) genes, and the *pmo*A/*mcr*A ratio (**c**) in the rhizosphere soil of different rice varieties at the tillering stage. Different letters in the same growth stage indicate significant differences among the rice varieties (*n* = 3, *p* < 0.05). The error bar indicates the standard deviation.

The abundance of methanogens and methanotrophs based on the *mcrA* and *pmoA* genes in the rhizosphere soil of five rice varieties at the tillering stage is shown in Figure 6. The abundance of methanogens in different rice varieties was significantly different (Longjing 20 > Suijing 18 > Longqing 31 > Longqing 32 > Longqing 31) (*p* < 0.05) (Figure 6a). The highest abundance of methanogens was in Longjing 20, reaching 6.89 ± 0.20 × 10^7^ copies·g^−1^ DWS, and was higher than that of the other varieties (21.8~64.9%) (Figure 6a). The highest abundance of methanotrophs was in Suijing 18, reaching 4.59 ± 0.76 × 10^7^ copies·g^−1^ DWS (Figure 6b). The abundance of methanotrophs in Suijing 18 was not significantly different from Longjing 31 and Longqing 32 (*p* > 0.05), but was significantly higher than Longqing 31 and Longjing 20 (*p* < 0.05) (Figure 6b). There was a significant difference in the *pmoA*/*mcrA* ratio (*p* < 0.05), where Longjing 31 was the highest, followed by Longqing 32 and Suijing 18, and Longqing 31 and Longjing 20 were the lowest (Figure 6c).

**Figure 7:**
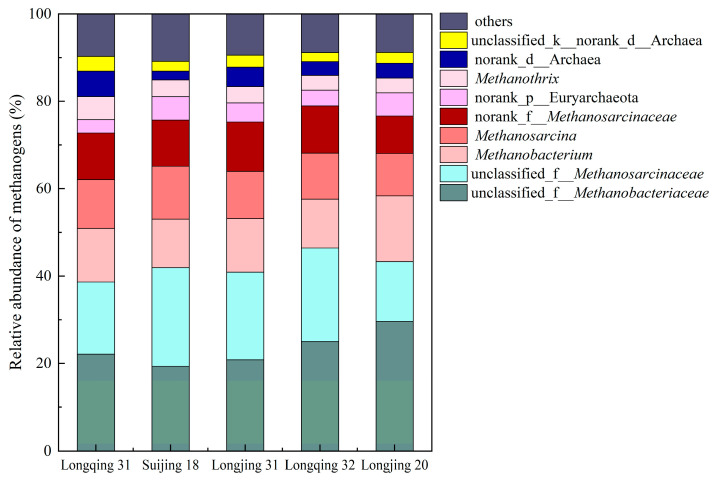
The community structure of methanogens in the rhizosphere soil of different rice varieties at the tillering stage.

At the genus level, the community structure of the methanogens and methanotrophs was classified. The methanogen community structure is depicted in Figure 7, where 70~77% of the methanogens are classified as unclassified_f__*Methanobacteriaceae*, unclassified_f__*Methanosarcinaceae*, *Methanobacterium, Methanosarcina,* and norank_f__*Methanosarcinaceae*. Among them, the unclassified_f__*Methanobacteriaceae* had the highest proportion, ranging from 19 to 29%.

**Figure 8:**
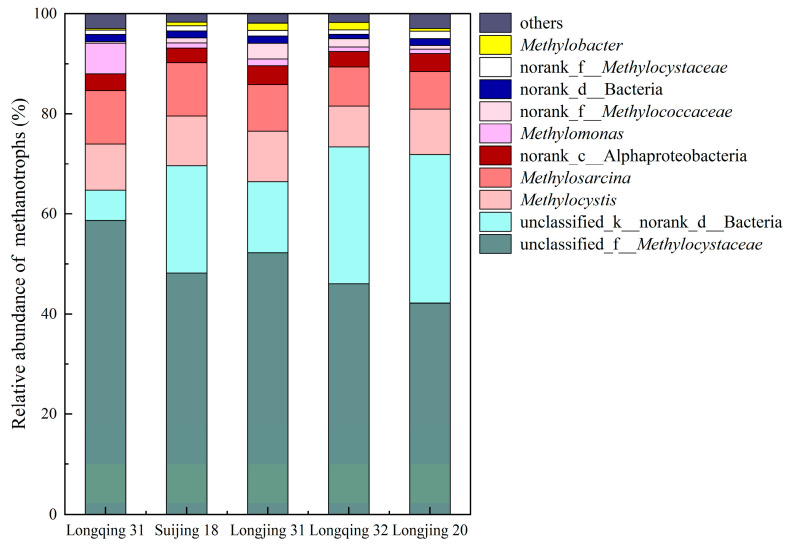
The community structure of methanotrophs in the rhizosphere soil of different rice varieties at the tillering stage.

The community structure of methanotrophs is shown in Figure 8, where unclassified_f__*Methylocystaceae* is the dominant genus, accounting for 42~59% of the methanotrophs, indicating the significant role it plays in methane oxidation. Longjing 20 had the lowest content of this methanotroph, while Longqing 31 had the highest. In contrast, the content of unclassified_k__norank_d__Bacteria showed an opposite effect, with Longjing 20 having the highest and Longqing 31 the lowest content.

**Figure 9:**
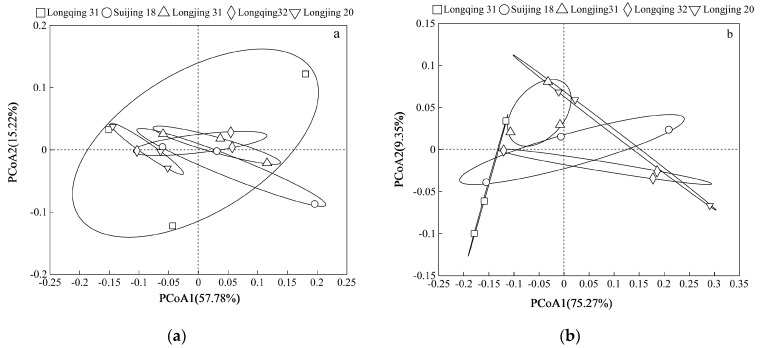
PCoA analysis of methanogen (**a**) and methanotroph (**b**) communities in rhizosphere soil of different rice varieties at tillering stage.

Principal coordinate analysis (PCoA) was performed on the community structure of methanogens and methanotrophs in different rice varieties at the genus level (Figure 9). For methanogens, the explanation rate of PCoA was 73%; the explanation rates of PCoA1 axis and PCoA2 were 57.78% and 15.22%, respectively (Figure 9a). For methanotrophs, the explanation rate of PCoA was 75.62%. The interpretation of the PCoA1 axis and the PCoA2 axis was 75.27% and 9.35%, respectively (Figure 9b), which means that these principal axes capture most of the variability in the data, so they can better characterize the relationship between methanogens and methanotrophs among rice varieties. The community of methanogens was relatively dense, indicating that the methanogen communities among the five rice varieties in this experiment were similar, while the community of methanotrophs was significantly separated along the 1 axis, indicating that there were significant differences in the community structure of methanotrophs among different rice varieties.

### 2.6. Effects of Rice Root Development and Rhizosphere Soil on CH_4_ Emissions from Paddy Fields

Correlation analysis was used to explore the correlation between the rice root development and rhizosphere soil-related indicators and the CH_4_ emissions from the paddy fields (Table 2). The CH_4_ emissions from paddy fields were significantly negatively correlated with *pmoA*/*mcrA*, the abundance of methanotrophs, ${NO}_{3}^{-}$–N, Eh, and rice root length (r = −0.68, −0.82, −0.80 and−0.60, *p* < 0.05). There were significant positive correlations with *mcrA*, ${NH}_{4}^{+}$–N, DOC, and MBC (r = 0.84, 0.75, 0.83, and 0.70, *p* < 0.05). The strongest correlation between all indicators and CH_4_ emissions is *pmoA*/*mcrA*.

Path analysis was used to reveal the role of these factors in influencing, directly or indirectly, the process of the CH_4_ emissions from the rice fields (Table 3). The results showed that the path analysis can effectively explain 78.8% of the impact, of which only the direct path coefficients of *mcrA* and *pmoA* are significant. The *mcrA* had a positive effect on CH_4_ emissions, while *pmoA* had a negative effect (the direct path coefficients are 0.91 and −0.47, respectively). The results show that methanotrophs and methanogens are the specific factors that directly affect CH_4_ emissions. Methanotrophs will inhibit CH_4_ emissions, while methanogens will promote CH_4_ emissions. From the indirect path coefficient, the root dry weight, root length, Eh, and DOC indirectly affected the methanogens and influence the CH_4_ emissions through this pathway. Conversely, ${NO}_{3}^{-}$–N negatively impacted methanotrophs and also played a role in indirectly shaping CH_4_ emissions. MBC affected both methanogens and methanotrophs, thereby promoting CH_4_ emissions from rice fields.

## 3. Discussion

### 3.1. CH_4_ Emission Rule in Paddy Field

In this study, the CH_4_ emission fluxes of different rice varieties showed similar trends during the whole growth period, and two significant emission peaks were recorded (Figure 1). The first peak appeared at the tillering stage, which may be due to the more vigorous respiration of rice at this stage, resulting in excessive CO_2_ being converted into CH_4_ [34]. In addition, the Eh values of the five rice varieties reached the lowest level during this growth period (Figure 3), and correlation analysis and path analysis showed that Eh affected CH_4_ emissions from paddy fields (Table 2 and Table 3). It indicated that Eh was closely related to CH_4_ emission from paddy fields. This is similar to the results of the previous study [35]. The second emission peak occurred at the heading–flowering stage; the rice produces a large amount of root exudate and litter. These organic substances provide abundant substrates for methanogens. This increases the abundance of methanogens and promotes CH_4_ emissions. As rice enters the maturity stage, the abundance of methanogens gradually decreases [32], leading to a subsequent decline in CH_4_ emission levels which are eventually stabilized. In terms of cumulative CH_4_ emissions, the highest proportion of emissions occurs during the tillering stage, indicating that regulating rice during this period could effectively reduce the CH_4_ emissions.

In this experiment, Longjing 31 had the highest *pmoA*/*mcrA* ratio and the lowest CH_4_ emissions. In contrast, Suijing 18 had the highest abundance of methanotrophs among the five varieties and also had a high abundance of methanogens (Figure 6). This suggested that, although Suijing 18 has strong CH_4_ oxidation capabilities, its high CH_4_ production capacity leads to relatively high net CH_4_ emissions. Furthermore, this indicated that the CH_4_ emissions from paddy fields are regulated by the dynamic balance between methanogens and methanotrophs. We also explored the microbial community factors that lead to differences in CH_4_ emissions between the low-emission CH_4_ variety Longjing 31 and the high-emission CH_4_ variety Longjing 20. The results showed that compared with Longjing 31, the proportion of unclassified_f__*Methanobacteriaceae* in methanogens of Longjing 20 was higher, and the proportion of unclassified_f__*Methylocystaceae* in methanotrophs was lower, indicating that these two bacteria would have a greater impact on CH_4_ emissions from paddy fields (Figure 7 and Figure 8). The methanogens are responsible for the decomposition of organic matter to produce CH_4_, while the methanotrophs oxidize CH_4_ released into the environment, thus affecting CH_4_ emissions. Path analysis further confirms that methanogens and methanotrophs are both direct factors affecting CH_4_ emissions (Table 3). This is because the *mcrA* gene encodes the key enzyme (methyl coenzyme-M reductase) in the methanogens that then catalyzes the final step of CH_4_ production, creating a significant impact on CH_4_ emissions [36]. The *pmoA* gene encodes particulate methane monooxygenase [37], which catalyzes the oxidation of CH_4_. Additionally, in this study, we observed differences in the composition of methanotroph communities among different rice varieties (Figure 9b), which is consistent with previous studies [38]. However, it is worth noting that the community structure of methanogens in different rice varieties is similar (Figure 9a). This phenomenon’s root cause lies in the clear biogeographic distribution of the methanogen community [39], and temperature is a critical environmental factor that shapes the unique community structure of methanogens in paddy fields over time [40]. Given that this study was conducted in the same geographical area, the community structure of methane-producing bacteria in each rice variety maintained a high degree of similarity. The results indicate that CH_4_ emissions in paddy fields can be effectively managed through the selection of appropriate rice varieties.

### 3.2. Effect of Rice Root Development on CH_4_ Emissions

The rice roots played a key role in regulating the CH_4_ emissions from paddy fields. The path analysis showed that the roots did not directly affect CH_4_ emissions; however, the roots did indirectly affect the CH_4_ emissions from paddy fields through the regulation of methanogens (Table 3). In this study, Longjing 31 showed the lowest total CH_4_ emissions characteristics (Figure 2), which may be attributed to its more prosperous root structure (Figure 4). The developed roots effectively improved the oxygen transport efficiency in the rhizosphere area, which in turn significantly increased the oxygen concentration. This effect was reflected in the Eh value [12]. Longjing 31 maintained a higher Eh value throughout the growth cycle (Figure 3). Given that methanogens are microorganisms in anaerobic environments, the increase in oxygen concentration effectively inhibits the activity and quantity of these microorganisms, thereby greatly reducing CH_4_ emissions [41,42]. Moreover, in the process of rice growth, the rice varieties with well-developed root systems will promote more photosynthetic products to be partially allocated to the panicle, reduce the production of carbon sources in the rice rhizosphere, limit the available substrates for methanogens, and further inhibit the production of CH_4_ [43]. In addition, in this study, it was also discovered that although rice roots affected the abundance of methanogens, there was no significant correlation between the abundance of methanotrophs. This may be because methanogens are extremely sensitive to oxygen; therefore, their activity is more easily influenced by the changes in oxygen concentration caused by rice roots. In this study, empirical support is provided for the strategy of selecting rice varieties with well-developed root systems as an effective approach for alleviating the excessive CH_4_ emissions from rice paddies.

### 3.3. Effects of Rice Rhizosphere Soil on CH_4_ Emissions

The results of this study showed that the rhizosphere soil had a significant effect on CH_4_ emissions by regulating the abundance of methanogens and methanotrophs. Inubushi et al. demonstrated a strong correlation between CH_4_ emissions and soil active organic carbon through their conducted experiments [44]. In our study, we discovered that DOC and MBC were both significantly positively correlated with CH_4_ emissions (*p* < 0.05). DOC mainly originates from root exudate and microbial degradation products and is considered to be a major carbon source for methanogens, promoting the production of CH_4_. Studies had shown that soil respiration rate is significantly positively correlated with MBC and DOC content, and CO_2_ is utilized by H_2_/CO_2_-dependent methanogens to contribute about 25–30% of CH_4_ production [45]. Meanwhile, several studies through a structural equation model had shown that MBC can promote the abundance of methanogens [46,47]. Li used chloroform fumigation to reduce the MBC levels by 43~87%, resulting in CH_4_ cumulative emissions decreasing to 1/352~1/1127 of the original amount [48]. This suggests that controlling the MBC levels might effectively regulate methanogen activity and thereby influence the CH_4_ emissions in paddy fields.

${NH}_{4}^{+}$–N and ${NO}_{3}^{-}$–N are important nitrogen forms in paddy soil, and they have a non-negligible effect on CH_4_ emissions from paddy fields. In this study, a significant positive correlation was identified between CH_4_ emissions and ${NH}_{4}^{+}$–N content, and a significant negative correlation was identified with ${NO}_{3}^{-}$–N content (Table 2). The path analysis (Table 3) showed that ${NH}_{4}^{+}$–N had a promotional effect on methanogens. This may be because all methanogens use ${NH}_{4}^{+}$–N as a nitrogen source [49]. Therefore, ${NH}_{4}^{+}$–N affects the content of methanogens and promotes CH_4_ production. However, ${NO}_{3}^{-}$–N can stimulate the abundance of these denitrifying methanotrophs, improve the oxidation capacity of CH_4_, and reduce the emission of CH_4_ [50,51]. In this study, the important effects of rhizosphere soil properties on CH_4_ emissions from paddy fields were revealed, and the indirect regulation of CH_4_ emissions through the management of the abundance of methanogens and methanotrophs was elucidated.

## 4. Materials and Methods

### 4.1. An Overview of the Experiment

**Figure 10:**
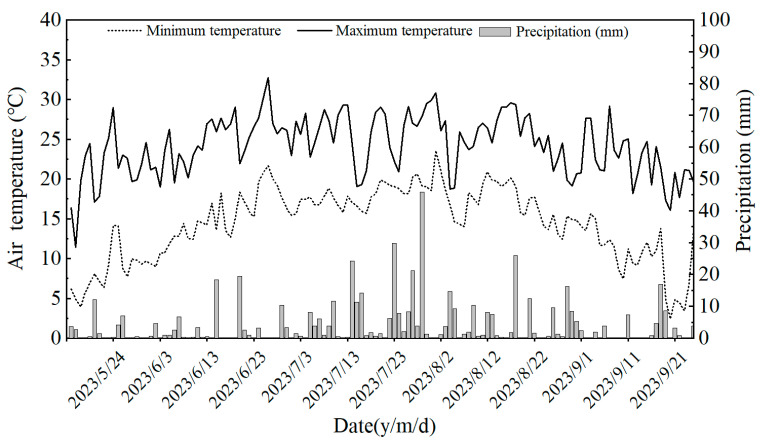
The air temperature and precipitation during the growth period of the rice.

The experiment was conducted in 2023 at the Qing’an State Key Station of Irrigation Experiment. The test site was situated in Heping Township, Suihua City (46°57′28″ N, 127°40′45″ E). According to the World Reference Base for Soil Resources 2022, black soil, classified as Phaeozems, falls under the Udic Humus Forms. This is the predominant soil type within the experimental area under study. The region in question is situated within the cold temperate continental monsoon climate zone. This region also exhibits an average annual duration of sunlight of approximately 2599 h, an average annual temperature of around 3.6 °C, a period of approximately 128 days free from the occurrence of frost, an average annual precipitation of approximately 574.4 mm, and an average annual evapotranspiration of approximately 1200~1600 mm. The specific meteorological conditions of the rice during the period from transplanting to maturity are illustrated in Figure 10. Prior to the commencement of the experiment, the five-point sampling technique was employed to collect field soil samples. Subsequent to this, an analysis of the soil’s principal physical and chemical attributes was conducted. The fundamental physical and chemical characteristics of the soil utilized in the experiment are detailed in Table 4.

### 4.2. Experimental Design

Five local rice varieties were selected for this experiment, namely Longqing 31, Suijing 18, Longjing 31, Longqing 32, and Longjing 20, which have a similar growth period. The experimental plots were transplanted on 22 May and the yield was measured on 24 September. The process of water management used conventional flooding irrigation. All experiments were conducted in three replicates. The layout of the CH_4_ emission measurement area was 15 hills × 10 rows, and the layout of the sample collection area was 15 hills × 20 rows. The dimensions of the transplanted seedlings were 30 × 13.3 cm, and the seedlings were cultivated manually to ensure that each hill contained three seedlings of similar size. In regard to fertilizer, the ratio of nitrogen fertilizer (46% N) was base fertilizer/tiller fertilizer/spike fertilizer = 4.5:2:3.5. The total amount of nitrogen fertilizer was 110 kg·hm^−2^. The phosphorus fertilizer (12% P_2_O_5_) was applied at a level of 45 kg·hm^−2^, and the potash fertilizer (60% K_2_O) was applied at a level of 80 kg·hm^−2^. The phosphorus fertilizer was applied in a single application prior to transplanting, while the potash fertilizer was divided into two applications, one before transplanting and the other when the rice reached the 8.5-leaf stage. The ratio of the two applications was 1:1.

### 4.3. Field Experiment Sampling and Measurement Methods

#### 4.3.1. CH_4_ Sampling and Measurement Methods

To quantify the emissions of CH_4_, a static box–gas chromatography method was employed [52]. Prior to the rice transplantation, stainless-steel bases were pre-buried within the designated measurement area. Each base was planted with six hills of rice, in accordance with the established experimental planting density. Then, three replicates were arranged in each experimental plot. The static box was constructed from acrylic board and wrapped in tinfoil, with light-blocking and heat-insulating effects on the outside of the box. The inside of the box was installed with a thermometer and a fan. The gas collection was performed at seven-day intervals following the rice transplantation. Sampling was carried out from 9:00 to 11:00. To ensure that the collected gas was not exchanged with the external space, before collecting the CH_4_, it was essential to fill the stainless-steel base with water to prevent any exchange of the gas with the external environment. At the four specified time points (0, 10, 20, and 30 min), the gas was collected from the static box using a gas collection bag. The temperature was recorded inside the static chamber after the experiment. Following the completion of the gas collection process, the samples were returned to the laboratory for quantitative analysis. This analysis was conducted using a gas chromatograph (GC-2010Plus, Shimadzu Corporation, Kyoto, Japan) for the purpose of determining the composition of the collected samples.

The CH_4_ emission fluxes and cumulative emissions were calculated as follows:

$$F=\rho h\frac{dc}{dt}\cdot \frac{273}{273+T}$$

where *F* is the CH_4_ emission fluxes (mg·m^−2^·h^−1^); ρ is the standard atmosphere condition density of a given gas (mg·m^−3^); *h* is the effective height of the entire collection device (m); $\frac{dc}{dt}$ is the slope of the curve depicting the concentration of gas within the box over time (mL·m^−3^·h^−1^); and T is the average temperature inside the box (°C).

$$E_{c}=\sum_{i=1}^{n}\frac{F_{i}+F_{i+1}}{2}\;(t_{i+1}{-t}_{i})\times \frac{24}{100}$$

where *Ec* is the cumulative CH_4_ emissions (kg·hm^−2^); *F_i_* and *F_i+_*_1_ are the CH_4_ emission fluxes at the *i*th and *i*+1th points (mg·m^−2^·h^−1^); and t_i+1_ − t_i_ is the number of days between *t*_i+1_ and *t* (d).

#### 4.3.2. Yield Measurement Methods

The yield of each rice variety was determined by the five-point sampling method. The rice yield was calculated according to the number of effective panicles, the number of filled grains per panicle, the 1000-grain weight, and the number of grains per panicle.

#### 4.3.3. Soil Sampling and Measurement

The Eh was monitored with an intelligent meter (YT-QX6530, Nanjing Institute of Soil Research, Nanjing, China). Rice rhizosphere soil was sampled using the root shaking method, as previously described [21]. The ammonium nitrogen (${NH}_{4}^{+}$–N) and nitrate nitrogen (${NO}_{3}^{-}$–N) contents were quantified through the application of the colorimetric method [53]. The soil samples were subjected to leaching, centrifugation, and filtration using a potassium chloride solution. Thereafter, the samples were analyzed using an AA3 flow analyzer (Seal Analytical GmbH, Norderstedt, Germany). The MBC contents were measured utilizing the chloroform fumigation method [54]. The soil samples were categorized into the two following groups: those that were fumigated with chloroform and those that were not. Following this, the carbon content of the two groups was ascertained using the volumetric method with potassium dichromate, after the samples were leached with K_2_SO_4_. The amount of DOC was that extracted from non-fumigated soil [55].

#### 4.3.4. Rice Root Sampling and Measurement

Taking the rice plant as the center, together with the rice, the surrounding 20 × 20 × 20 cm soil blocks were excavated and cleaned with a hydropneumatic rinsing device. The samples were then placed in a dryer set at 70 °C and dried thoroughly until the weight remained constant, thus ensuring an accurate measurement of the dry mass of the roots. Another fresh rice root system was taken as previously described and then floated in a shallow water tray for the analysis of the rice root development using a root scanner (Expression 12000XL, Seiko Epson Corp., Suwa, Japan) and a root analyzer (WinRhizo LA2400, Regend Instruments Inc., Québec City, QC, Canada).

#### 4.3.5. DNA Extraction and Real-Time PCR

In this study, considering the high CH_4_ emission characteristics during the tillering stage of rice, the abundance and community composition of the methanogens and methanotrophs were measured in various rice varieties during the tillering stage. The experiment was conducted by the Shanghai Majorbio Bio-Pharm Technology Co., Ltd. (Shanghai, China). The DNA was extracted from the soil using YH-soil, the FastPure Soil DNA Isolation Kit (MJYH, Shanghai, China). The abundance of methanotrophs and methanogens in soil samples was examined using quantitative real-time PCR. The specific primer sequences used were as follows [56]: A189F: GGNGACTGGGACTTCTGG and mb661R: CCGGMGCAACGTCYTTACC for *pmoA*, and MLfF: GGTGGTGTMGGATTCACACARTAYGCWACAGC and MLrR: TTCATTGCR TAGTTWGGRTAGTT for *mcrA*. In this phase of the experiment, one pair of primers was designed for each of the two genes. The results of the PCR demonstrated that the electrophoretic gelograms of each primer exhibited a single band under specific conditions. This finding indicated that the primers could be utilized in the subsequent experiments. The constructed plasmids were identified via sequencing, and the OD_260_ values of the plasmids were subsequently measured using a UV spectrophotometer (NanoDrop2000; Thermo Fisher Scientific, Waltham, MA, USA). The data were then converted into copies/µL employing the following formula: A 10-fold gradient dilution of the constructed plasmids was performed, with 90 µL of dilution and 10 µL of plasmid. Then, the 10^−2^~10^−7^ dilutions of the *pmoA* standard and the 10^−1^~10^−7^ dilutions of the *mcrA* standard were selected for the preparation of standard curves through the pre-tests. The PCR cycling conditions were as follows: the initial steps took place at 95 °C for 3 min, melting at 95 °C for 5 s, annealing at 55 °C for 30 s, and were finally extended at 72 °C for 1 min. After completing the aforementioned steps, the 96-well plates containing the samples were placed in an ABI 7300 real-time fluorescence quantitative PCR instrument (Applied Biosystems, Waltham, MA, USA) for the reaction.

#### 4.3.6. Determination of Community Composition of Methanogens and Methanotrophs

In this experiment, functional gene sequencing was employed. The same primer sequences as mentioned in Section 4.3.5 were used for amplification. The PCR products were purified, and a paired-end (PE) library was constructed for high-throughput sequencing on the Illumina MiSeq PE300 platform by the Shanghai Majorbio Bio-Pharm Technology Co., Ltd. The PCR products from the same samples were mixed and detected using 2% agarose gel electrophoresis. The PCR products were then gel-purified using the AxyPrepDNA Gel Extraction Kit (Axygen Biosciences, Union City, CA, USA) based on the preliminary quantification results from the electrophoresis. The quantification was performed using the QuantiFluo^TM^-ST blue fluorescence quantitation system (Promega, Madison, WI, USA), and the PCR products were mixed in proportions based on the required sequencing amounts for each sample. Following this, the library construction and sequencing for the Illumina platform were conducted. The paired-end reads obtained from the Illumina sequencing were first ligated based on their overlap relationships, and the sequences were subjected to quality control and filtering. After distinguishing the samples, operational taxonomic units (OTUs) were clustered and taxonomically classified. Based on the OTU results, various diversity indices could be analyzed, and the sequencing depth could be assessed. With the taxonomic information, the community structure could be statistically analyzed at different taxonomic levels. On this basis, the community compositions of the multiple samples could be compared and analyzed.

#### 4.3.7. A Statistical Analysis of the Data

SPSS v. 22 (IBM Inc., Armonk, NY, USA) was used for the statistical analysis of the data; one-way analysis of variance (ANOVA) was applied to assess the differences among varieties for each indicator, which was statistically significant at the level of *p* < 0.05. And, depending on whether the data were homogeneous or not, either Tukey’s test or Games—Howell’s test was used to assess the significance of differences. The relationships among the indicators were evaluated through correlation analysis, and the potential mechanisms of the rice root system and its rhizosphere soil on CH_4_ emissions were analyzed using path analysis. This approach allowed us to quantify the contribution of each physiological indicator to methane emissions. The mapping was conducted using the Origin 2021 (Origin Lab corporation, Northampton, MA, USA) software.

## 5. Conclusions

In the black soil region of Northeast China, an important rice production base, it is of great significance to study the relationship between CH_4_ and paddy fields to realize green agriculture and ensure food security. In this study, field experiments examining five rice varieties in Northeast China were carried out to reveal the regulation provided by rice root development and rhizosphere soil on CH_4_ emissions from paddy fields. The results indicated that different rice varieties exhibit varying CH_4_ emissions, demonstrating the feasibility of reducing CH_4_ emissions through variety selection. Methanogens and methanotrophs are the factors that directly affect the CH_4_ emissions from paddy fields. CH_4_ emissions depend on the dynamic balance of methanogens and methanotrophs, and the rice roots and rhizosphere soil regulate these two microorganisms to indirectly control CH_4_ emissions. Additionally, this study also identified a significant negative correlation between rice root development status (root length and root dry weight) and CH_4_ emissions. The carbon and nitrogen components in the rhizosphere soil simultaneously and significantly affect the CH_4_ emissions; the ${NH}_{4}^{+}$–N, DOC, and MBC contents promote the CH_4_ emissions; meanwhile, the ${NO}_{3}^{-}$–N contents inhibit CH_4_ emissions. And the Eh value is significantly negatively correlated with CH_4_ emissions. In summary, in the black soil area of Northeast China, selecting rice varieties with good root development and strengthening rhizosphere soil management are effective ways of reducing CH_4_ emissions and promoting agricultural sustainability.

## Figures and Tables

**Table 1:** Cumulative CH_4_ emissions, rice yield, and CH_4_ emissions per yield of each rice variety.

Rice Varieties	Cumulative CH_4_ Emissions (kg hm^−2^ ± s.d.)	Yield (t·hm^−2^ ± s.d.)	CH_4_ Emissions per Yield (t·t^−1^ ± s.d.)
Longqing 31	398.3 ± 17.5 b	8.65 ± 0.34 b	0.046 ± 0.0021 b
Suijing 18	325.7 ± 25.1 c	9.63 ± 0.28 ab	0.034 ± 0.0016 c
Longjing 31	256.3 ± 21.1 d	10.19 ± 0.37 a	0.025 ± 0.0012 e
Longqing 32	285.0 ± 19.7 cd	9.58 ± 0.14 ab	0.030 ± 0.0016 d
Longjing 20	457.5 ± 18.9 a	7.55 ± 0.19 c	0.061 ± 0.0010 a

Note: different letters indicate significant differences among rice varieties (*n* = 3, *p* < 0.05).

**Table 2:** The correlation analysis of CH_4_ emissions with rice root development characteristics and soil factors.

	CH_4_ Emissions	*mcrA*	*pmoA*	*pmoA/mcrA*	${NH}_{4}^{+}$–N	${NO}_{3}^{-}$–N	DOC	MBC	Root Dryweight	Root Length	Eh
CH_4_ emissions	1										
*mcrA*	0.84 **	1									
*pmoA*	−0.68 **	−0.24	1								
*pmoA/mcrA*	−0.92 **	−0.78 **	0.71 **	1							
${NH}_{4}^{+}$–N	0.75 **	0.84 **	−0.30	−0.67 **	1						
${\mathrm{NO}}_{3}^{-}$–N	−0.82 **	−0.50	0.83 **	0.92 **	−0.54 *	1					
DOC	0.83 **	0.77 **	−0.44	−0.78 **	0.65 **	−0.61 *	1				
MBC	0.70 **	0.58 *	−0.55 *	−0.65 **	0.70 **	−0.79 **	0.49 *	1			
Root dryweight	−0.48 *	−0.67 **	0.04	0.47	−0.73 **	0.34	−0.27	−0.39	1		
Root length	−0.60 **	−0.69 **	0.26	0.60*	−0.36	0.24	−0.71 **	−0.22	0.03	1	
Eh	−0.80 **	−0.72 **	0.58 *	0.94 **	−0.66 **	0.88 **	−0.77 **	−0.68 *	0.44	0.54 *	1

Note: *, ** indicate significant correlation at the 0.05 and 0.01 levels, respectively.

**Table 3:** Path analysis of total cumulative CH_4_ emissions by rice root system and soil components.

Independent Variable	Direct Path Coefficient	Indirect Path Coefficient	
		*mcrA*→CH_4_	*pmoA*→CH_4_
Root dry weight	−0.09	−0.61 *	0.00
Root length	0.17	−0.63 *	−0.21
${NH}_{4}^{+}$–N	−0.23	0.76 *	0.14
${NO}_{3}^{-}$–N	−0.15	−0.46	−0.39 *
DOC	0.27	0.70 *	0.21
MBC	0.07	0.53 *	0.26 *
Eh	0.23	−0.66*	−0.27
*mcrA*	0.91 *		
*pmoA*	−0.47 *		

Note: R^2^ = 0.955, *p* < 0.05, e = $\sqrt{1-R^{2}}$ = 21.2%. This indicates that path analysis can effectively explain 78.8% of the impact. * indicates significant differences at the level of 0.05.

**Table 4:** The properties of the experimental soil.

Soil Properties	
pH (H_2_O)	6.4
Organic matter (g·kg^−1^)	40.9
Alkaline N (mg·kg^−1^)	177.8
Available P (mg·kg^−1^)	35.2
Available K (mg·kg^−1^)	125.1
Total porosity (%)	61.8
C/N Ratio	13.5
Soil texture	Sandy clay loam

## Data Availability

The data presented in this study are available on request from the corresponding author.
